# Correction to: Sequence-encoded conformational biases correlate with self-assembly modes of intrinsically disordered proteins

**DOI:** 10.1093/pnasnexus/pgag331

**Published:** 2026-10-06

This is a correction to: Ryoga Kobayashi, Norio Yoshida, Yohei Miyanoiri, Hideki Nakamura, Miu Ekari, Emi Sakamoto, Misato Tsutsumi, Kayo Imamura, Takashi S Kodama, Hidehito Tochio, Naotaka Sekiyama, Sequence-encoded conformational biases correlate with self-assembly modes of intrinsically disordered proteins, *PNAS Nexus*, Volume 5, Issue 7, July 2026, pgag189, https://doi.org/10.1093/pnasnexus/pgag189

In the originally published version of this article, a funding statement was inadvertently missed. The sentence “This work was supported by the Japan Society for the Promotion of Science (JSPS) KAKENHI grant number JP25H00845 (to H.T.)” has been added to the funding section.

